# Correction: A framework for identifying calcium accumulation problem in cropland: Integrating field surveys, legacy soil map, and machine learning models

**DOI:** 10.1371/journal.pone.0334107

**Published:** 2025-10-14

**Authors:** Xingjie Yin, Haile Zhao, Yuchao Luo, Yuling Jin, Zhihua Pan, Wenting Liu, Pingli An

All authors except the corresponding author, Xingjie Yin, Haile Zhao, Yuchao Luo, Yuling Jin, Zhihua Pan, and Wenting Liu, should not have been attributed equal contribution to this work.
