# Supplementary figures and images for: A framework for identifying calcium accumulation problem in cropland: Integrating field surveys, legacy soil map, and machine learning models
Source: PLoS One. 2025 May 30;20(5):e0325076. doi: 10.1371/journal.pone.0325076 (PMC12124509; doi:10.1371/journal.pone.0325076)

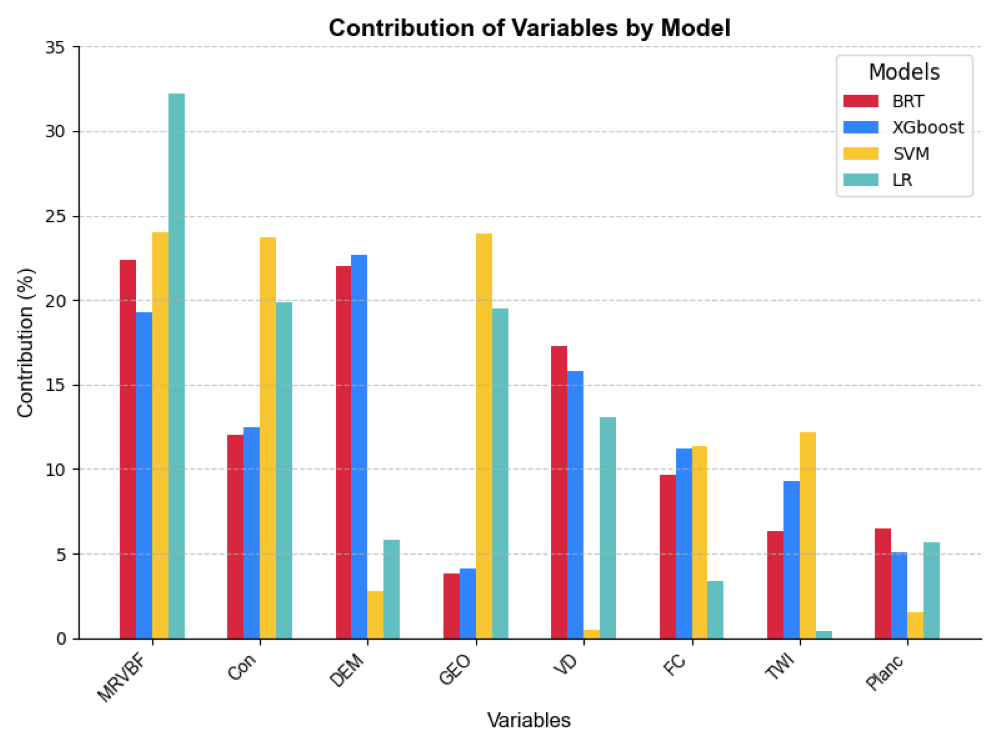

Supplement: S1 Fig — (TIF) [file pone.0325076.s001.tif]
