# Supplementary material for: A framework for identifying calcium accumulation problem in cropland: Integrating field surveys, legacy soil map, and machine learning models
Source: PLoS One. 2025 May 30;20(5):e0325076. doi: 10.1371/journal.pone.0325076 (PMC12124509; doi:10.1371/journal.pone.0325076)
Supplement: Attachment — Submitted filename: 1_1_Response to Reviewers.docx [file pone.0325076.s004.docx]

Dear Editors,

We appreciate the opportunity to revise our manuscript **[A framework for identifying calcium accumulation problem in cropland: Integrating field surveys, legacy soil map, and machine learning models]** and would like to sincerely thank you for your constructive feedback. Below, we provide a detailed response to each of the editorial comments and outline the revisions or explanations accordingly.

**1. Ethics Approval**

We confirm that our study does not require ethics approval, as it does not involve human or animal subjects, and no personal or sensitive information was collected. To comply with the editorial request, we have obtained a formal explanation letter from our affiliated institution—College of Land Science and Technology, China Agricultural University.

The statement was reviewed and approved by:

1) Dr. Haishuang Jia, Director of Scientific Research and Social Services,
2) Ms. Zhaohong Cao, Deputy Party Secretary and Vice Dean,
3) Ms. Xiaohe Yu, Chief Officer of General Affairs.

We have uploaded the scanned letter, including the official stamp, in both Chinese and English versions, under “Other” files for your reference.

**2. Data Availability**

We regret that we are unable to fully share our key dataset due to confidentiality agreements and protection of residents' geospatial privacy. These restrictions are in compliance with local data governance regulations.

However, all openly available data sources used in the study have been listed in the manuscript. Further explanation regarding the data sharing constraints and the contact information for data requests has been added to the Data Availability statement.

**3. Code Availability**

We respectfully clarify that our modeling code is not fully original and was developed based on the widely referenced method by Elith et al. As such, it is not applicable to publish this code as a standalone contribution.

Furthermore, the key modeling parameters, data processing steps, and algorithm settings have been clearly outlined in Table 4 of the manuscript for full transparency. We believe this provides sufficient reproducibility for readers. Should any issues arise, we remain available for clarification.

**4. Map and Satellite Image Copyright**

We have addressed the copyright concerns in the following ways:

1) Figure 1: The original basemap has been removed.
2) Figures 1, 3, 4, 5, and 6: All geographic boundary data (including China’s administrative boundaries) were obtained from the National Geomatics Center of China, a publicly accessible and free source.

We have updated the captions and methods section to reflect the data source and have included the map approval number GS(2024)0650 in the manuscript.

**5. Field Permits and Ethics Statement Placement**

We have revised the Methods section to clarify that no field permit was required, as the study area is public and no environmental disturbance or restricted activity was involved.

**6. Funding Statement**

As requested, we have added the following standard disclaimer to the “Acknowledgments” of the manuscript:“The funders had no role in study design, data collection and analysis, decision to publish, or preparation of the manuscript.”

**7. File Uploads and Formatting**

We have prepared and uploaded the following revised materials according to PLOS ONE requirements:

1) Response to Reviewers (this document)
2) Revised Manuscript with Track Changes
3) Clean Manuscript (without track changes)
4) Ethics Statement Document and Data availability (uploaded under “Other” files)

We hope these revisions and clarifications adequately address the editorial concerns. Please feel free to contact us if further information is required.

Sincerely,

[Pingli An]

On behalf of all co-authors

[College of Land Science and Technology, China Agricultural University]

Email: [anpl@cau.edu.cn]

****Reviewer#1**：**

1. **What are the key factors contributing to calcium accumulation in croplands?**

Thank you very much for your insightful question. Identifying the key factors contributing to calcium accumulation is indeed crucial and deserves a clear explanation in both the Introduction and Methods sections. Your comment prompted us to revisit and revise the relevant content accordingly.

Calcium accumulation problems (CAP) in soils primarily arise from the excessive buildup of pedogenic carbonates (PC). Therefore, understanding the drivers of PC accumulation is essential for addressing the mechanisms behind CAP. Previous studies have extensively examined PC formation, revealing that climate, topography, and human activities are the dominant influencing factors. Under the influence of these drivers, PC undergoes repeated processes of dissolution, translocation, and re-precipitation, eventually forming various morphological features in the soil.

In the context of our study, CAP is also controlled by these primary factors—climate, topography, and human activities. Given the specific characteristics of the region under investigation, we further emphasize the role of parent material, which provides a sufficient source of calcium carbonate for accumulation. While climate sets the general environmental background, its variation is relatively limited within the study area. Human activity, although important, is constrained by data availability. Therefore, we focus on topographic factors, which strongly influence moisture distribution at the soil surface under similar climatic conditions. These micro-environmental differences play a critical role in the formation and spatial differentiation of CAP. ***The detailed descriptions are as follows:***

***Line 49-57: “PC is deposited in various forms (including earthworm biospheroliths, rhizoliths, pseudomycels, nodules, coatings and calcrete) through the dissolution, movement and re-precipitation of calcium carbonate[3]. PC is further redistributed under the influence of climate[4–6], topography[7–10], human activities[11–14], etc. A sediment layer of a certain depth is formed through eluviation and illuviation of PC, which varies with precipitation[11,15]. The amount of PC increases with the land use changes to cropland[11,13,16]and gradually accumulates in the shallow layers[17,18]. Irrigation can eluviate PC to deeper layers[17,19], while in arid regions without additional water inputs, PC tends to accumulate in the shallow layers of croplands[20,21].”***

***Line 102-107: “CAP is defined as a soil characteristic that negatively impacts crops by degrading the physical and chemical properties of the soil due to the accumulation of carbonates. PC is the primary component of these accumulated carbonates, with calcium carbonate being the main constituent of PC[3]. The essence of CAP lies in the accumulation of PC, therefore factors influencing PC formation are key contributors to CAP, like parent material, topography, climate and human activity, etc.”***

**2. How does the study integrate field surveys with machine learning models?**

Thank you very much for this important question. The integration of field survey results with machine learning models is indeed one of the core aspects of our study. However, we acknowledge that this was not clearly articulated in the original manuscript. Thanks to your careful review, we have revised the relevant sections to better explain this integration.

In brief, the field survey component includes both questionnaire responses and soil sampling data. The questionnaire results helped validate our understanding of the CAP phenomenon and provided theoretical support for the construction of the machine learning models. They also offered contextual insights into local perceptions and experiences, which reinforced the rationale for selecting certain modelling variables. On the other hand, the soil sampling results were used to verify both the LSM (legacy soil map) and the predictions generated by the machine learning models. This validation process strengthened the reliability of our findings and enhanced the overall credibility of the study. ***The detailed descriptions are as follows:***

***Line 281-284: “These findings suggest that CAP is more likely to occur on sloping terrains, indicating that topographical factors play a significant role in CAP development. In addition to providing supplementary data to the construction of machine learning models, the findings of the survey provide a realistic basis for the interpretability of modelling results.”***

3. **What role do legacy soil maps play in identifying calcium accumulation?**

Thank you for your valuable question. The use of legacy soil map (LSM) in identifying calcium accumulation problems (CAP) is a central element of our study. We initially addressed the role of LSM in the "LSM Sampling" section, but your insightful comment prompted us to review and revise this part to better emphasize their significance.

In our study, LSM serve as the foundation for constructing the CAP dataset. Specifically, LSM provide the “Y” variable—i.e., whether a CAP exists or not—and the spatial locations of sample points obtained through random sampling. These sampled locations were then used to extract relevant independent variables, enabling the construction of the full dataset for model training and analysis. ***The detailed descriptions are as follows:***

***Line 123-128: “The county-level LSM scale is 1:50,000, providing an accurate spatial distribution of various soil types for sampling. Before LSM sampling, we validated the soil map using data from 33 filed-dug soil profiles. (Fig 2) illustrates some of the profiles, with the phenomenon of CAP, forming points, blocks, and surfaces. For each profile, we recorded the actual observed soil type, coordinates, and CAP labeling information (S1 Table). The LSM was compared to the actual soil types, yielding an overall accuracy of 94%, confirming the reliability of the LSM.”***

***Line 131-135: “When "carbonate deposits" were observed in a typical soil profile, it is assumed that CAP was present in the corresponding soil type. (Fig 3a) illustrates the distribution of soil types in southern Aohan Banner, where 628 of 1106 cinnamon soil polygons exhibit CAP. The number of sampling points per cinnamon soil polygon was determined based on area as weight and randomly sampled. A total of 10,000 sample points were allocated, some of which are shown in (Fig 3b).”***

4. **Which machine learning models were used in the framework**

Thank you for your insightful question. The application of machine learning models is a key component of our approach to identifying calcium accumulation problems (CAP). Model selection is crucial, as it directly affects the accuracy and robustness of the results. Although we briefly outlined our model choices in the “Model Selection” section, we acknowledge that our original explanation lacked clarity. In response to your comment, we have revised and expanded this section to provide a clearer rationale.

In selecting the models, we considered both linear and non-linear relationships among the terrain variables. To capture complex, non-linear interactions, we applied three widely used machine learning models with varying levels of complexity: XGBoost, Boosted Regression Trees (BRT), and Support Vector Machines (SVM). Additionally, we included logistic regression to account for potential linear relationships, providing a baseline for comparison.

This combination allowed us to explore how different models interpret the relationship between predictors and CAP occurrence, ensuring a more comprehensive and interpretable modeling framework. ***The detailed descriptions are as follows:***

***Line 169-172: “For the purpose of resolving linear or non-linear relationships between terrain variables, we chose three common models (XGBoost, BRT and SVM, with decreasing model complexity in that order) to account for non-linear relationships between terrain variables, and Logistic regression to account for linear relationships between terrain variables (Table 4).”***

5. **What soil properties were considered in the study**

Thank you for your thoughtful question. Considering soil properties is indeed essential for identifying calcium accumulation problems (CAP). In our study, some soil properties were directly or indirectly taken into account, though we did not explicitly elaborate on them in the original manuscript. We appreciate your attention to this aspect, which prompted us to revise and supplement the relevant content.

Specifically, we considered key soil physical and chemical properties such as calcium carbonate content and soil pore size, as these characteristics influence the formation and accumulation of calcium in the soil. Among these, only calcium carbonate content was explicitly used in our framework to determine the presence of CAP, serving as a criterion in labeling the data.

However, due to the limited spatial variability and availability of other soil property data, we did not include them as independent variables in the machine learning models. Instead, we relied on topographic factors, which reflect the spatial heterogeneity of the landscape and indirectly capture variations in soil properties relevant to CAP. ***The detailed descriptions are as follows:***

***Line 158-162: “In summary, spatial dissimilarity of variables is key to identification. Thus, we only wish to consider the soil physical and chemical properties, such as calcium carbonate content, soil pore size, etc. These properties manifest themselves differently in different terrains. We therefore identified CAP by considering topographic factors to indirectly consider the combined expression of soil properties.”***

6. **How does calcium accumulation affect crop productivity**

Thank you for this meaningful question. We briefly mentioned the impact of calcium accumulation problems (CAP) on crop productivity in the introduction; however, your comment prompted us to revisit and improve the clarity of our explanation.

CAP affects crop productivity by altering the soil's physical and chemical properties. On the physical side, excessive calcium carbonate accumulation can clog soil pores and reduce water infiltration, which limits the availability of water to plant roots. On the chemical side, high levels of carbonate can interfere with the absorption of essential micronutrients, thereby impairing plant growth and yield. ***The detailed descriptions are as follows:***

***Line 58-62: “Calcium accumulation problem (CAP) caused by excessive PC accumulation, which affects the physical, chemical and biological properties of the soil[22], and thus indirectly affects crop productivity. Such as clogging soil pores[23], weakening inter-root water infiltration and movement[24], reducing plant utilization of micronutrients[15], and threatening sustainable agricultural development.”***

7. **What were the major data sources for the analysis**

Thank you for your insightful question. Data sources are indeed foundational to our study. We revisited the “Data” section and improved the clarity of the descriptions regarding the datasets used in our framework. The major data sources include: field survey data (questionnaires), the Third National Soil Survey of China, local soil archive records (soil monographs), and topographic data. Each of these datasets contributed to different parts of the model construction and validation process. We also supplemented additional details to clarify specific sources, such as the data used for assessing soil erosion intensity. ***The detailed descriptions are as follows:***

***Line 235-240: “Soil erosion intensity. To assess the impact of soil erosion on CAP, this study incorporates soil erosion intensity as a variable in the model. According to the "Soil Erosion Classification and Grading Standards," soil erosion is classified into hydraulic, wind, and freeze-thaw erosion types. Erosion intensity is further categorized into slight, moderate, strong, and extreme based on the average erosion modulus (t/km²×a: <1000, 2500, 5000, 8000, 15000) and the average loss thickness (mm/a: <0.74, 1.9, 3.7, 5.9, 11.1). This data can be found in*** [***www.resdc.cn***](http://www.resdc.cn) ***for free.”***

8. **What are the advantages of using machine learning for soil assessment?**

Thank you for your valuable question. This is indeed a critical aspect of our study, as the use of machine learning plays a central role in soil assessment and the identification of calcium accumulation problems (CAP). Clarifying the advantages of machine learning in this context is essential for understanding the methodological strengths of our approach.

Although we discussed the predictive utility of combining legacy soil maps (LSM) and ML models in the first part of the *Discussion*, we realized that we had not clearly explained the specific advantages of machine learning techniques. ***The detailed descriptions are as follows:***

***Line 383-385: “Overall, machine learning models can capture non-linear relationships in soil assessment and have strong generalisation capabilities. It also allows for theoretical analyses through model interpretation. And, it has considerable advantages for scaled tasks.”***

9. **What were the key findings of the study**

Thank you for your insightful question. The key findings of our study can be summarized into four main aspects: (1) the conceptual analysis of CAP formation mechanisms, (2) a comparative evaluation of different machine learning models, (3) the spatial prediction of CAP occurrence, and (4) the interpretability and analysis of the model outputs. Among these, the most critical contribution lies in demonstrating the feasibility of using LSM and machine learning for soil assessment, especially in identifying CAP. ***The detailed descriptions are as follows:***

***Line 516-519: “The results show that tree-based models, particularly the Boosted Regression Trees (BRT), provide higher accuracy and better mapping performance compared to XGBoost. This approach to constructing soil characteristic datasets demonstrates significant feasibility, offering a cost-effective alternative for regions with limited soil profile data.”***

10. **How can this framework help in sustainable soil management**

Thank you for your thoughtful question. We agree that the ultimate goal of understanding, identifying, and analyzing CAP should be to support practical applications and contribute to sustainable soil management. In the “Recommendations for mitigation strategies” section, we proposed a few potential approaches to mitigate CAP, including deep tillage and improved irrigation practices. More importantly, our proposed framework offers a cost-effective means for identifying the spatial distribution of CAP or other soil characteristics, especially in data-scarce regions. By integrating LSM, field surveys, and machine learning models, this framework can help land managers understand the underlying causes of CAP and make informed, evidence-based decisions to guide land-use planning and differentiated soil management. ***The detailed descriptions are as follows:***

***Line 494-497: “Our paper creates a framework for identifying CAP on cropland, using LSM and combining both survey and machine learning methods. It enables governments and managers to optimise land-use planning and to implement differentiated management policies. The framework provides a theoretical basis for the sustainable management of local soils.”***

****Reviewer#2**：**

1. **Lack of novelty- novelty of the manuscript is missing**

We appreciate the reviewer’s comment regarding the novelty of our manuscript. We respectfully disagree that the manuscript lacks novelty. In this study, the novelty lies in the integration of LSM with machine learning to identify CAP. To the best of our knowledge, This work has not been extensively studied, especially the spatial identification of calcium accumulation problems in agricultural fields. Nevertheless, we understand the importance of clearly stating the novelty. Therefore, we have revised the result section to better highlight the innovative aspects. We hope this clarifies the novelty of our study. ***The detailed descriptions are as follows:***

***Line 511-515: “In this paper, a novel approach to constructing datasets is used, i.e., LSM sampling data rather than relying on traditional survey data. This approach improves data accessibility and provides a low-cost solution for soil characterization data construction for regions lacking profile data. Moreover, the relationship between CAP and terrain features was revealed by an effective machine learning model.”***

2. **The selection of machine learning models (BRY, XGBoost, SVM, and LR) was not well justified**

Thank you for this insightful comment. We agree that the original explanation for model selection lacked sufficient detail. In response, we have revised the “Model selection” section to clarify the rationale behind choosing Boosted Regression Trees (BRT), XGBoost, Support Vector Machine (SVM), and Logistic Regression (LR).

These models were selected based on their ability to capture different types of relationships between variables and their varying levels of model complexity. Specifically, XGBoost, BRT, and SVM were used to explore non-linear relationships between terrain variables, while LR was adopted to account for potential linear relationships. This tiered approach also enables comparison across models of varying interpretability and generalization capabilities. ***The detailed descriptions are as follows:***

***Line 169-172: “For the purpose of resolving linear or non-linear relationships between terrain variables, we chose three common models (XGBoost, BRT and SVM, with decreasing model complexity in that order) to account for non-linear relationships between terrain variables, and Logistic regression to account for linear relationships between terrain variables (Table 4).”***

3. **The study was conducted in a specific region (Aohan banner, Chifeng City) - Compare with any other region**

Thank you for pointing this out. Aohan Banner was selected as the study area because it is representative of the broader cinnamon soil zone within the Liaohe River Basin. This region experiences widespread environmental stressors—such as drought, low rainfall, and severe soil erosion—which make it highly suitable for investigating CAP.

Due to data availability constraints, we focused our analysis on Aohan Banner. However, we recognize the importance of enhancing regional applicability. To address this, we have provided a more detailed description of Aohan Banner in the “Materials and Methods” section and expanded the discussion by comparing our findings with studies from other regions. This allows us to place our results within a broader geographical context and demonstrate the generalizability of the identified patterns. ***The detailed descriptions are as follows:***

***Line 82-86: “There are 47,000 km2 cropland in cinnamon soil area from Liaohe Plain, where there are widespread problems such as drought, low rainfall, serious soil erosion, and obvious calcium deposits. Aohan banner is a typical area in Liaohe Plain, located in the southeastern quadrant of Chifeng City, Inner Mongolia (Fig 1b). Aohan Banner's landscape features a gentle and uneven slope, descending from the southeast to the northwest.”***

***Line 424-434: “Similar patterns are evident in studies from other regions(3,9,20,55). Dunling wang initiated a study on the landscape distribution of PC in chernozemic soil in the Southeastern Saskatchewan region. Whose results indicated that strongly sedimentary topographic environments, such as slopes and depressions, facilitate the removal of shallow PC[20]. This occurs because runoff from adjacent slopes creates wetter soil conditions compared to upslope areas, promoting PC leaching in the shallow layers[55]. G. Jacks studied the relationship between calcareous horizons and hillside landscapes in southern India[8]. The results show that, due to the movement of carbonate solutions, it is more likely to form calcrete at deeper levels. Additionally, Some studies in Mexico have indicated that soils on both sides of ridges, slopes, or in runoff positions have shallower PC accumulation due to reduced water infiltration[7,10]. Such shallow accumulation of PC is closely associated with CAP formation.”***

4. **Suggested to include additional evaluation metrics and external validation datasets to strengthen the conclusions**

Thank you for this valuable suggestion. We fully agree that incorporating external datasets is essential to validating the reliability of the predictive results. In response, we introduced an independent dataset on soil erosion intensity to externally validate our model outputs. This approach helps strengthen the conclusions of the study by linking CAP probability with an independently measured indicator of soil degradation. ***The detailed descriptions are as follows:***

***Line 441-446: “For this extended view, we introduce an external dataset to validate it. As the intensity of soil erosion increases, the probability of CAP also gradually rises (Fig 10.). The analysis reveals that as the erosion intensity increases, the probability of CAP becomes more stable and higher. Although extreme erosion has fewer samples, making its statistical representation less robust, areas with slight erosion consistently exhibit lower probabilities of CAP. This trend aligns with the study's main findings, suggesting that an increase in erosion intensity leads to a higher probability of CAP.”***

**Fig 10. Relationship between predicted values and soil water erosion intensity with area distribution.**

The bar chart represents the area distribution of different soil erosion intensities in cinnamon soil cropland; the box-and-line plot represents the CAP probability distribution of different soil erosion intensities in cinnamon soil cropland

5. **It is noted that the discussion section did not adequately acknowledge the limitations of the study**

Thank you for pointing this out. We agree that the discussion of the study's limitations was initially insufficient. In the revised manuscript, we have added a dedicated paragraph in the discussion section to explicitly address the major limitations of our work. ***The detailed descriptions are as follows:***

***Line 498-508: “However, there are some limitations to our study. To a large extent, the LSM is our basic data. The accuracy of the LSM and the level of detail of the information available are related to the precision of the identification results. Up to now, the results of the third national soil census have not been fully popularized, and most of the soil data still remain from the second soil census. This leads to the lack of accuracy of the results. The results of the third soil census are more detailed and their distribution is more accurate with the addition of emerging technologies. Therefore, in the future, when the third soil census results are popularized, it can effectively improve the accuracy of the results in the framework of this paper. Aohan banner is a extended area of Liaohe basin. Most of all, Aohan banner is the typical area in North cinnamon soil zone. But, Limited by the difficulty of obtaining high-precision soil data over a wide area, we have only conducted research in Aohan banner, with a view to providing a reference for other regions. ”***

6. **This manuscript does not provide strong recommendations for practical mitigation strategies**

Thank you for this constructive suggestion. While our study did identify potential mitigation strategies based on field observations, we acknowledge that these recommendations were not sufficiently discussed. We have now expanded the relevant section to highlight feasible mitigation options—particularly deep tillage and irrigation—and discussed their potential application based on site-specific conditions. ***The detailed descriptions are as follows:***

***Line 486-492: “In summary, irrigation and deep tillage may be effective methods to address CAP in cinnamon soil cropland. While leaching the shallow soil carbonates, these methods can also improve the physical and chemical properties of the soil and enhance the plow layer. How to implement solutions locally needs to be based on actual conditions, such as the availability of external water sources, the depth of PC accumulation, and the topographical conditions of the cropland. Based on these conditions, the management characteristics of different regions should be clarified to facilitate further field experiments to determine the practical effects of these methods.”***

7. **Some sections of the manuscript were noted as difficult to follow due to unclear phrasing and grammatical errors - change it.**

We thank the reviewer for pointing out the language and clarity issues in our manuscript. In response, we have conducted a comprehensive revision of the entire text. All grammatical errors and unclear sentences have been carefully corrected. We also revised the phrasing and structure in several paragraphs to ensure better coherence and readability. The revised version has been thoroughly proofread, and we believe the overall clarity and presentation of the manuscript have been significantly improved. We sincerely appreciate your feedback, which helped us enhance the quality of the work.
